# Ethical principles for artificial intelligence in education

**DOI:** 10.1007/s10639-022-11316-w

**Published:** 2022-10-13

**Authors:** Andy Nguyen, Ha Ngan Ngo, Yvonne Hong, Belle Dang, Bich-Phuong Thi Nguyen

**Affiliations:** 1 Learning & Educational Technology Research Unit (LET), University of Oulu, Oulu, Finland; 2 Faculty of Education, Victoria University of Wellington, Wellington, New Zealand; 3 School of Information Management, Victoria University of Wellington, Wellington, New Zealand; 4 Faculty of English Language Teacher Education, VNU University of Languages and International Studies, Hanoi, Vietnam

**Keywords:** Artificial Intelligence, AIED, Ethics, Policies, Privacy

## Abstract

The advancement of artificial intelligence in education (AIED) has the potential to transform the educational landscape and influence the role of all involved stakeholders. In recent years, the applications of AIED have been gradually adopted to progress our understanding of students’ learning and enhance learning performance and experience. However, the adoption of AIED has led to increasing ethical risks and concerns regarding several aspects such as personal data and learner autonomy. Despite the recent announcement of guidelines for ethical and trustworthy AIED, the debate revolves around the key principles underpinning ethical AIED. This paper aims to explore whether there is a global consensus on ethical AIED by mapping and analyzing international organizations’ current policies and guidelines. In this paper, we first introduce the opportunities offered by AI in education and potential ethical issues. Then, thematic analysis was conducted to conceptualize and establish a set of ethical principles by examining and synthesizing relevant ethical policies and guidelines for AIED. We discuss each principle and associated implications for relevant educational stakeholders, including students, teachers, technology developers, policymakers, and institutional decision-makers. The proposed set of ethical principles is expected to serve as a framework to inform and guide educational stakeholders in the development and deployment of ethical and trustworthy AIED as well as catalyze future development of related impact studies in the field.

## Introduction

The application of artificial intelligence (AI) in education has been featured as one of the most pivotal developments of the century (Becker et al., 2018; Seldon with Abidoye, 2018). Despite the rapid growth of AI for education (AIED) and the surge in its demands under the COVID-19 impacts, little is known about what ethical principles should be in guiding the design, development, and deployment of ethical and trustworthy AI in education. And even if those are addressed, the depth and breadth to which contemporary ethical and regulatory frameworks are able to capture the impacts of AI evolvement remain unfolded.

The complexity and “intelligence” of this technology have led to potentially extensive ethical threats that trigger a pressing need for risk-intensive procedures to ensure the quality of delivery. Indeed, a sense of flexibility that acknowledges human values within the developing momentum of AI is vital to fostering sustainable innovations. In the wake of such demand, UNESCO launched global standards for AI ethics which were agreed and signed by its 193 member countries on November 25, 2021. The document, whilst recognizing the “profound and dynamic” influences of AI, also highlights related flourishing dangers to the cultural, social, and ecological diversity (United Nations Educational, Scientific and Cultural Organization [UNESCO], 2021). Notably, it stipulates a universal framework of values for ethics which provides stakeholder-driven guidelines in adopting AI. This historic cross-border agreement marks the globally significant role of ethics in AI; however, it provides a relatively generic framework across disciplines and settings. In fact, for the development and governance of AI technologies, neither laissez-faire nor one-size-fits-all approach is adequate and appropriate across contexts. In the literature, ongoing debates regarding ethics of data exploitation in decision making and interventions occur cross-disciplines (Jalal et al., 2021; Farris, 2021; medical care as in Reddy et al., 2020) or human resources management, as in Tambe et al., (2019); sports performance analysis as in Araújo et al., (2021). Recently, researchers and international organizations have specifically examined the ethics of AI in education (Holmes et al., 2021). Despite there being some overlaps and common agreements among these ethical guidelines and reports, no previous study has systematically assessed a global consensus on ethics for AIED.

Our study attempts to fill these gaps by examining and matching ethical guidelines and reports from UNESCO Ethics AI (Ad Hoc Expert Group [AHEG], 2020), UNESCO Education & AI (Miao et al., 2021), Beijing Consensus (UNESCO, 2019), OECD (Organization for Economic Co-operation and Development [OECD], 2021), European Commission (2019), and European Parliament Report AI Education (2021). We sought to prescribe a set of ethical principles for trustworthy AIED based on the thematic analysis results. The establishment of unified ethical principles for AIED gives the research agenda in this domain a new opportunity to meet the demands of a widespread digitalization of education.

This paper is organized as follows. We first introduce a holistic picture of AI in education and present the emerging opportunities. Then, we provide a critical review of the extant literature on ethical issues of AI in education. Next, we present the thematic analysis results of the relevant ethical guidelines and reports for AIED then discuss the implications for associated educational stakeholders. Finally, we conclude by highlighting the significance of ethics in the contemporary discussion of education through which propose several key ethical principles that underpin ethical and trustworthy AIED.

## Opportunities of artificial intelligence in education

The penetration of AI in every sphere of educational practices has undeniably filtered teachers’ and students’ personal and professional development with numerous opportunities (Xu & Ouyang, 2021; Ouyang et al., 2022). Existing literature has witnessed a wide diversity of perspectives on the use of AI in education, ranging from the non-teaching aspects (e.g., timetabling, resource allocation, student tracking, provision of information about students to their parents/guardians (reports) to the personalization of teaching and learning (tailored design and marking of assessments, curriculum and AI apps that support learners, or locate changes in learner engagement during foreign language learning (Fahimirad & Kotamjani, 2018; Luckin, 2017; Reiss, 2021; Skinner et al., 2019). Hwang et al., (2020) identified four key roles of AI in education driven by an applications-based perspective that espouses the position of AI as an intelligent tutor, tutee, learning tool/partner, or policy-making advisor.

AIED is seen as an influential tool to empower new paradigms of instruction, technology advancement, and innovations in educational research that are deemed unfeasible in the conventional classroom settings, for instance, the implementation of artificial neural networks, machine learning or CALL (Computer-Assisted Language Learning) in formal, non-formal and informal learning scenarios (Holmes et al., 2019; Hwang et al., 2020). It enables computer-assisted collaborative learning or asynchronous discussion groups, allows cost-wise personalized learning through a navigation system underpinned by algorithms (Nye, 2015), promoted by the use of automated assessment,facial recognition systems, and predictive analytics (Akgun & Greenhow, 2021). Hence, there are growing evidence for the roles of AIED to “foster a transformation of knowledge, cognition, and culture” (Hwang et al., 2020, p.1). However, the implementation of AIED has faced several challenges related to ethical concerns and justification.Although recent attempts have been made to provide ethical guidelines for AIED, there remains the question of a global consensus and standard guidelines for AIED. As the regulation and ethical consensus of these technologies is needed for utilizing their various capabilities in education, this paper sought to offer an integrated overview of ethical guidelines for AIED.

## Ethical issues of AI in education

Despite its capability to revolutionize education, numerous challenges also linger for researchers and practitioners who are involved in associated activities or systems (Kay & Kummerfeld, 2019) as AIED is, by nature, a “highly technology-dependent and cross-disciplinary field” (Hwang et al., 2020, p.2). At a global level, UNESCO (2019) pinpointed six challenges in achieving sustainable development of AIED: comprehensive public policy, inclusion and equity in AIED, preparing teachers for AI-powered education,preparing AI to understand education, developing quality and inclusive data systems, making research on AIED significant, ensuring ethics and transparency in data collection, use, and dissemination. At the individual level, challenges range from critical societal drawbacks such as systemic bias, discrimination, inequality for marginalized groups of students, and xenophobia (Hwang et al., 2020) to thorny ethical issues relating to privacy and bias in data collection and processing (Holmes et al., 2021). In fact, the widespread ramifications of AIED have also led to emerging concerns over the negative realities that it brings, such as the widening gaps of inequalities among learners’ commercialization of education, or the home-school divide in education (Reiss, 2021). AI may become pervasive in every sense where those involved may be exposed to risks without being aware of them, and the situation can be even intensified under the ongoing impacts of the COVID-19 pandemic (Borenstein & Howard, 2021). Such obstacles essentialize an urgent demand to induct and acquaint teachers and students with the ethical concerns surrounding AIED and how to navigate them.

Furthermore, AIED also carries ethical implications and privacy risks which call for critical attention to differentiate between doing ethical things and doing things ethically (Holmes et al., 2021), or as in the words of Russell and Norvig (2002) “all AI researchers should be concerned with the ethical implications of their work” (p. 1020). Indeed, a proliferation of studies has revealed the emergence of contrasting ethical themes relating to general AI and AIED, most of which are associated with the liability of data across settings, such as in higher education (Zawacki-Richter et al., 2019), K-12 (Holstein et al., 2019), schools (Luckin, 2017), and subjects (Hwang & Tu, 2021). These covered the issues of informed consent, privacy breach, biased data assumption, fairness, accountability, and statistical apophenia. Others also question the impacts of AI-related fields such as surveillance and consent, learner privacy (Sacharidis et al., 2020), identity configuration, user confidentiality, integrity, and inclusiveness (Deshpande et al., 2017). Another stream of discussion has been drawn upon the ethics of data designated for educational use and analytics learning (e.g., Kay & Kummerfeld 2019; Kitto & Knight, 2019; Slade & Prinsloo, 2013). These incorporate the spheres of data interpretation and management, different perspective on the data usage, and the power relation among involved stakeholders such as students, teachers, and the educational objectives (Slade & Prinsloo, 2013). Other ethical issues for AIED include the problems with data collection, restricted availability of data sources, bias and representation, data ownership and control, data autonomy, AIED systems, and human agency (Akgun & Greenhow, 2021; Miao et al., 2021). That said, it is crucial to fully comprehend these values and principals before making ethically and accountability-driven decisions, and being aware of possible, even unexpected outcomes in education.

Although recent work has attempted to establish different ethical frameworks for general AI use (e.g., Ashok et al., 2022), ethical and privacy issues are suggested to be contextualized (Ifenthaler & Schumacher, 2016), hence the prior guidelines established in other disciplines might not be appropriate for education. The contextual approach to the ethical design and use of AIED could play an essential role in addressing the issues of ethical and privacy concerns in education context. Prior research has emphasised the importance of the sociotechnical context configured by educational technology and educations practices in ethical considerations (Kitto & Knight, 2019). The understanding of ethics and privacy from various perspectives could promote the design of ethical and trustworthy AIED and the adoption of such systems. Furthermore, we extended the ethical view from published studies reviewed by Ashok et al., (2022) to the policies and guidelines proposed by the international organizations such as UNESCO, OECD, and European Union. The consensus assessment of policies and guidelines would inform a comprehensive and integrated instructions for different stakeholders in adopting AIED. This contributes to establishing a common ground and solid foundation for further development and implementation of AIED.

## Ethical principles for artificial intelligence in education (AIED)

There are continued calls for substantial ethical guidelines and open communications with beneficiaries: educators, students, parents, AI developers, and policymakers (Berendt et al., 2020; Nigam et al., 2021; Hagendorff, 2020) stated that more emphasis is necessary to enforce ethical guidelines for AI systems to better align with societal values. Safeguard measures and human oversight are required to oversee how these AI systems are designed, how they function and evolve. The knowledge of behavioral science, equipped with self-awareness and empathy at the fore, is argued to intrinsically motivate AI developers to develop more trustworthy and responsible AI (Dhanrajani, 2018).

We conducted thematic analysis on relevant ethical guidelines and reports related to AIED found from international organizations, including UNESCO Ethics AI (AHEG, 2020), UNESCO Education & AI (Miao et al., 2021), Beijing Consensus (UNESCO, 2019), OECD (Organization for Economic Co-operation and Development, 2021), European Commission (2019), and European Parliament Report AI Education (2021). The paper focused on identifying and developing a set of main principle themes by using inductive analysis, based on Braun & Clarke (2012)’s thematic analysis process. The analysis consists of initial familiarization with the ethical guidelines and reports. This involved re-reading of reports and noting down patterns, such as similar use of words, points of discussion, and definitions. This is followed by an open coding approach where the terms and definitions are meaningfully categorized, followed by labeling each category with a code. This resulted in a total of 39 codes. Next, these codes were examined and collated into patterns of broader meaning, resulting in 7 themes (i.e. principles). The coding and themes generation process was conducted iteratively, where a researcher-researcher corroboration method was also in place to ensure the reliability and validity (Patton, 2015) of the proposed principles and corresponding code mapping in Table 1.

**Table 1:** Ethical Principles for Artificial Intelligence in Education

Ethical Principles for AIED	Codes	Sources					
	**General**	UNESCO Ethic AI 2020 (Draft)	UNESCO Education & AI (2021)	Beijing Consensus (2019)	OECD (2021)	European Commission (2019)	European Parliament Report AI Education (2021)
Governance & Stewardship	Governance & Stewardship	✔	✔	✔	✔	✔	✔
	Multistakeholder	✔	✔		✔	✔	
	Interdisciplinary planning		✔				
	International Cooperation	✔			✔		
	Monitoring & Evaluation	✔	✔		✔	✔	✔
Transparency & Accountability	Transparency	✔	✔	✔	✔	✔	✔
	Explicability					✔	
	Explainability	✔	✔		✔	✔	✔
	Responsibility	✔					
	Accountability	✔				✔	
	Auditability	✔	✔	✔		✔	✔
Sustainability & Proportionality	Sustainability	✔	✔		✔	✔	✔
	Environment	✔			✔	✔	
	Local Alignment	✔	✔	✔			
	Proportionality	✔	✔	✔		✔	
	Economy & Labour	✔	✔		✔	✔	✔
	Lifelong learning		✔	✔			
Privacy	Data Privacy	✔	✔	✔	✔	✔	✔
	Children’s Privacy		✔				✔
Security & Safety	Data governance					✔	✔
	Safety	✔			✔	✔	✔
	Robustness			✔	✔	✔	✔
	Prevention of harm	✔	✔	✔		✔	✔
	Security				✔	✔	✔
Inclusiveness	Inclusiveness	✔	✔	✔	✔	✔	✔
	Accessibility		✔			✔	✔
	Diversity	✔				✔	✔
	Integrity of data					✔	✔
	Non-discriminate Data	✔				✔	✔
	Algorithms biases		✔			✔	✔
	Fairness	✔	✔	✔	✔	✔	✔
	Gender equality	✔	✔	✔		✔	✔
Human-centered AIED	Human Oversight	✔	✔		✔	✔	✔
	Human-centric/centered		✔	✔	✔	✔	✔
	Human Rights	✔				✔	
	Human Dignity	✔				✔	
	Human (Learner) Agency		✔	✔		✔	
	Human Autonomy	✔			✔	✔	✔
	Teacher Role		✔	✔			

### Principle of governance and stewardship

A recurring theme across AI policies is the issue of governance and stewardship of AIED (Ashok et al., 2022). For example, the 2021 UNESCO Education & AI 2021 asserted the need to “set up a system wide organizational structure for policy governance and coordination” (Miao et al., 2021, p32). This is further acknowledged in other papers such as OECD (2021, p.4) recommendation for “Principles for responsible stewardship of trustworthy AI”. AIED governance and stewardship declares and manages how AI should be employed in education and relevant mechanisms to assure the compatibility between the role of the technology being deployed and its designed purposes, to optimize educational stakeholders’ needs and benefits. AI principle of governance has been formally defined as “the practice of establishing and implementing policies, procedures and standards for the proper development, use and management of the infosphere.” (Floridi, 2018, p.3). Meanwhile, AI stewardship could be defined as ethics embodied in the careful and responsible management of the design and use of AIED. Although governance and stewardship have been mentioned in most ethical guidelines and policies for AIED, these issues have been surprisingly disregarded from many contemporary ethical debates in the literature (Ashok et al., 2022). While governance refers to “a structure or pattern, stewardship is an activity” (Greer, 2018, p.42). In other words, taking action on issues such as building capacity or developing transparency from a long list of policies can be seen as good stewardship or setting up a better governance. According to OECD Principles for responsible stewardship of trustworthy AI (OECD, 2021), there are five complementary principles relevant to all stakeholders: (i) inclusive growth, sustainable development and well-being; (ii) human-centred values and fairness; (iii) transparency and explainability; (iv) robustness, security and safety; and (v) accountability. While the first and second principles sought to attain the inclusiveness and human-centredness in AIED, the later three OECD principles share several common intersections with data ethics and physical safety in using AIED. Accordingly, we propose that the governance and stewardship of AIED should accomplish all ethical aspects of relevant domains.

Principle of governance and stewardship: The governance and stewardship of AIED should carefully take into account the interdisciplinary and multi-stakeholder perspectives as well as all ethical considerations from relevant domains, including but not limited to data ethics, learning analytics ethics, computational ethics, human rights, and inclusiveness.

The consideration of soft and hard ethics from relevant domains in the governance and stewardship of AIED is critical for the ethical design and use of trustworthy AIED and enhancing its societal implications.

### Principle of transparency and accountability

Data ethics emphasized the need for transparency in data usage in AIED (Larsson & Heintz, 2020). AI tools have been gradually applied quite extensively in education to enhance learning and teaching practices (Wang & Cheng, 2021), but the challenge remains unaddressed regarding the transparency of the data generated. Cope & Kalantzis (2019) highlighted that this ethical principle is essential to teachers and students as data visualization represents learner behavior, and accentuates additional support that educators could provide. It should be noted that the transparency lies in what the data itself is, where it is collected, what it shows, what happens to it, and how it is used (Digital Curation Centre, 2020). These questions could be answered once data ownership, accessibility, and explainability are sustained.

The notion of data ownership, by nature, is a matter of transparency and fairness (Remian, 2019), dealing with who owns and is entitled to the rights to access the personal data of learners. Although technically speaking, consent may often be given to data collectors, whether the data usage intrudes on learners’ privacy has long remained controversial. A valid argument aligned with the integrity of the motive for data collection could be proposed, in which the ownership should be granted to students themselves. Indeed, students are those providing data, thereby having the rights to own and control how data should be used to benefit their own learning (Holmes et al., 2021). Meanwhile, there comes a plausible claim about the rights of institutions to access and use student data since interactions and performance of learners, in essence, are recorded using a structured learning system provided by these educational institutions.

The concept of explainability in AI and data is closely linked to the transparency of the AI system and data generated. Indeed, data should feature the ability to explain some predictions from a technical viewpoint of a particular human. AI explainability underscores the insights in how AI systems functioned and made a decision should be well informed and explicable to the stakeholders, though the explicability relies on their technical expertise and role (Kazim & Koshiyama, 2021; UNESCO, 2019). The opaque nature of AI often poses many challenges for stakeholders to fathom the logic of this “black box” behind its decision-making. For instance, the absence of explainability could result in teachers being unable to use AIED effectively and timely detect the problems related to students’ behavior and learning performance (Remian, 2019). Therefore, this ethical concern centers on the intelligibility of the operation and outcomes of AI educational systems.

Principle of transparency in data and algorithms: The process of collecting, analyzing, and reporting data should be transparent with informed consent and clarity of data ownership, accessibility, and the purposes for how data will be used. The AI algorithms should be explainable and justifiable for specific educational purposes.

The transparency of AIED has been highlighted in several ethical guidelines, including the European Commission’s ethics guidelines for trustworthy AI (2019), European Parliament (2021), UNESCO Education & AI (Miao et al., 2021), Beijing Consensus UNESCO (2019), and OECD’s Principles for responsible stewardship of trustworthy AI (2021). However, the components and descriptions of transparency vary among these reports and guidelines. For instance, while the European Commission (2019, p.18) explained it as “closely linked with the principle of explicability and encompasses transparency of elements relevant to an AI system: the data, the system and the business model”, UNESCO 2020 Draft points to its association “to adequate responsibility and accountability measure” (AHEG, 2020, p.10). Alongside the transparency in data and algorithms, transparency should be of utmost significance to all AIED regulations.

Principle of Transparency in Regulation: The process of establishing, conducting, monitoring, and controlling regulations of AIED should be transparent, traceable, explainable, and communicable in an open and clear manner with clarity of regulatory roles, accessibility, responsibilities, the purposes for how AI will be developed and used, and under which conditions. Additionally, the regulation of AIED should be transparent in its auditability, and it also links with the next ethical principle of regulatory accountability.

Principle of accountability: The regulation of AIED should explicitly address acknowledgment and responsibility for each stakeholder’s actions involved in the design and use of AIED, including auditability, minimization, and reporting of negative side effects, trade-offs, and compensation.

The accountability of AIED relates to the concept of “responsible AI” that features the ethical practice of designing, developing, and implementing AI with good intentions to empower relevant stakeholders and society fairly. Though ‘responsible AI’ has become increasingly popular, the terms accountability and responsibility are rarely defined (Jobin et al., 2019). Nevertheless, it is commonly referred to as acting with integrity and clearly determining the attribution of responsibility and legal liability with careful consideration of potentially harmful factors. AI has been questioned over whether it should be held accountable in a human-like manner, or whether humans should always be the sole actors responsible for AI as technological artifacts. In conjunction with human-centered AIED that encouraged human oversight over AI, we recommended the latter case that educational stakeholders should always be the responsible ones for AIED. Furthermore, some AI policies have highlighted that regulation of AIED should step beyond the scope of individual and organizational accountability to also consider sustainability and proportionality (AHEG, 2020).

### Principle of sustainability and proportionality

Similar to other technology advances, the development and deployment of AI should also take into account environmental concerns to the extent that is referenced (AHEG, 2020; OECD, 2021). Particularly, sustainability calls for the design, development, and use of AIED to consider optimizing energy efficiency and minimizing its ecological footprint (European Commission, 2019). Accordingly, regulations of AIED are required to create policies ensuring these considerations are accomplished throughout the processes of developing and deploying AIED. Moreover, regulation of AIED must consider other sustainable domains, including economic and societal aspects such as employability, culture, and politics (European Parliament, 2021).

Principle of sustainability and proportionality: AIED must be designed, developed, and used in a justifiable way that they would not disrupt the environment, world economy, and society, such as the labor market, culture, and politics.

For instance, regulation of AIED should consider ensuring policies supporting accountability of potential job losses and to leverage challenges as an opportunity for innovation (UNESCO, 2019). Careful deliberations of sustainability and proportionality will make AIED more approachable and beneficial to all.

### Principle of privacy

Personal privacy also emerged as a critical ethical concern in the implementation of AIED. Privacy, by nature, could be defined as “the right to be left alone”, which underscores the right of having personal information being protected (Muller, 2020). This digital revolution in education, particularly the use of AI and learning analytics in the field of education, entails a massive amount of personal data generated, captured, and analyzed to optimize learning experiences (Tzimas & Demetriadis, 2021; Pardo & Siemens, 2014). The personal data of teachers and learners may run the risk of privacy breaches. For instance, in respect of agent-based personalized education, personal information of learning performance accumulated in the past could be utilized for future prediction. However, this is considered against the will of many students (Li, 2007).

To protect and support the right of learners’ privacy and social well-being while learning in the context of increasingly knowledgeable machines and computer agents, AIED developers need to assess the views of teachers and students to decide how AI should be deployed in the classroom (Miao et al., 2021). For instance, an ethical concern may arise from a real-time facial expression recognition system used to predict the affective state (e.g. Jian-Ming Sun et al., 2008) or attendance of the learners without their consent (e.g. Pattnaik & Mohanty 2020). Developers and educators should embed transparency and visibility to AIED-related threats while explaining potential ramifications to students’ learning, careers, and social lives. The objective is to cultivate trust among learners and provide them with insights to leverage their skills across contexts while maintaining control of their respective data and digital identities (Jobin et al., 2019).

Principle of privacy: AIED must ensure well-informed consent from the user and maintain the confidentiality of the users’ information, both when they provide information and when the system collects information about them.

In most cases, when AIED tools are used to engage users in a particular learning activity, users are assumed to give consent, by which they would agree on terms of use of technology and how their personal data is collected, managed, and processed. Aligned with the principle of transparency and accountability, consent must be well informed as a pragmatic approach to building trust among students since the consent demonstrates their ease with the use of data by teachers to enhance their own learning performance (Li et al., 2021; Sedenberg & Hoffmann, 2016) also highlighted the significance of this consent to show respect towards students and reinforce their autonomy and freedom of choice. Once data is garnered, questions arise about how data management works, where and how long their personal information should be stored, and to whom the rights of accessibility should be granted (Corrin et al., 2019).

### Principle of Security and Safety

One of the main functions of educational learning systems is to collect data of users, from which predictions about the learning behaviors and performance of users will be made. However, it is inevitable to envisage a scenario when the data is probably manipulated or corrupted by another party, or even worse, by cybercriminals.

Principle of Security: AIED should be designed and implemented in a manner that ensures the solution is robust enough to safeguard and protect data effectively from cybercrimes, data breaches and corruption threats, ensuring the privacy and security of sensitive information.

The concept of incorruptibility in AIED traces its root from incorruptibility in AI, or robustness against malicious manipulation by external factors. Bostrom & Yudkowsky (2014) pointed out that AI systems must be “robust against human adversaries deliberately searching for exploitable flaws in the algorithm” (p. 317). Therefore, it can be stated that the incorruptible nature and integrity of the data go hand in hand with data security. It is essential to protect the personal data of stakeholders, including students, teachers, and schools, to prevent any misuse or violation. The protection of data privacy and security is even more essential in the current context of normalizing virtual learning, and it requires concerted effort and the self-awareness of all the stakeholders.

Whereas learning analytics are governed by data ethics, many AIs are forms of intelligence expressed by some artifact (Bryson & Theodorou, 2019) that interacts with humans at various levels, such as robots and self-driving cars (Manoharan, 2019; O’Sullivan et al., 2019). This raises a universe of technical safety concerns regarding AI operation throughout its lifecycle in normal use, especially in harsh conditions or where other agents (both human and artificial) can interfere with the system.

Principle of Safety: AIED systems to be designed, developed, and deployed in a risk-management approach so that users are protected from unintended and unexpected harm, and that fatalities are mitigated.

As a result, it is pivotal that AIED developers take great care to design, train, pilot test, and validate the safety of AI systems (Leslie, 2019). Multistakeholder groups, including product developers, educators, and public authorities, should establish appropriate oversight, assessment, and due diligence mechanisms to ensure accountability and robustness throughout the AI lifecycle (AHEG, 2020). This group should produce detailed guidelines and ensure that AI users (educators and learners) receive adequate training to operate the system safely within the defined environment.

### Principle of inclusiveness

Previous ethical discourse suggested that AI systems should contribute to global justice and be equally accessible to all (European Commission, 2018). Accessibility is vital to allow society to gain significant benefits from these systems. The exclusion of any individual is a violation of human rights. It is, hence, paramount that accessibility entails affordability, user-friendly designs catering to individuals of different demographics, cultures, and particularly those with disabilities (Kazim & Koshiyama, 2021). As highlighted in the European Commission Report 2021, inclusion and fairness of access to AI-powered education stress the basic needs and availability for internet coverage, followed by next-generation digital infrastructure.

Principle of Inclusiveness in Accessibility: AIED design, development, and deployment must take into account the infrastructure, equipment, skills, and societal acceptance that will accommodate a wide range of individuals in the intended region, allowing equitable access and use of AIED.

The current digital gap evidently widens after COVID-19, where countries with poor infrastructure hamper their aspirations of thriving in digitalization (Palomares et al., 2021). Furthermore, the fundamental lack of access to technologies, such as students from socially disadvantaged backgrounds not owning personal digital devices (Sá et al., 2021), calls for collective discussions with all educational stakeholders on the aspects of inclusion in AIED (i.e. addressing the lack of opportunities, resource sharing efforts to counter areas suffering from deprivation of learning resources, and knocking down discriminatory structures) to reduce educational inequities (Office of the High Commissioner for Human Rights, 2019).

Another aspect of inclusiveness is non-discrimination or unbiased AI algorithms. Quality education is fundamental in fostering a flourishing society, where all learners are viewed equally regardless of their gender, race, beliefs, sexual orientation, and any other conditions or circumstances (Palomares et al., 2021). AIED design requires careful considerations to avoid discrimination against certain groups, as AIED relies on and will only be as good as its trained data. Hence, it is crucial that AI developers take precautions by training the AIED with comprehensive and diverse data to reduce instances where the AIED would manifest a particular bias (Hogenhout, 2021) and violate the non-maleficence principle.

Principle of Inclusiveness in Data and Algorithms: AIED design, development, and deployment must apply non-discrimination and unbiased data and algorithms to ensure fairness and equality among different groups of beneficiaries.

Data quality plays a crucial role in determining whether AIED could make valid and unbiased decisions since bias manifests itself in the AIED system with the biased training data (Borgesius, 2018; Digital Curation Centre, 2020). Several aspects of biased data relating to gender, race, ethnicity, and special learning needs. An illuminating example in language education technology is given by West-Smith et al., (2018) that input data in the form of rubric writing and scoring system may place a constraint on the task choice and writing styles of students. Thus, there is a need for bias-free data in AIED to avoid biased algorithms.

### Principle of human-centered AIED

In recognition of autonomy as a modern moral and political value (Calvo et al., 2020), the development and regulation of AIED need to adopt a human-centric approach that safeguards and empowers human autonomy This principle emphasizes the importance of supporting learners in developing their own potential (Miao et al., 2021; UNESCO, 2019).

Principle of Human-Centred AIED: The goal of AIED should be to complement and enhance human cognitive, social, and cultural capabilities while preserving meaningful opportunities for freedom of choice, securing human control over AI-based work processes.

Human autonomy, according to Deci and Ryan (2020), refers to the capacity to live one’s life according to one’s own motivation that is not the result of deception or manipulation. AI assistants today serve a variety of functions, generally intending to provide and assist individuals with some recommendations. In a sense, these can be considered external factors that affect an individual’s cognitive bias and emotions undermining or manipulating one’s intrinsic motivation (Vesnic-Alujevic et al., 2020). The design and operation of AI must thus, avoid misleading information, compromising users’ autonomy in developing independent thoughts, or negatively affecting users’ emotions and social well-being.

Research and development on AIED must avoid algorithms and wordings that serve as computational propaganda (Brundage et al., 2018; Nobre, 2020) in the form of automated feedback, learning assessment, and suggestions. This is particularly pertinent in an educational context, where many users are children and young people, constituting a vulnerable group that deserves special care and protection (European Parliament, 2021). There should be training programs supporting educators to gain the required skills to implement AIED. They should be able to adapt, filter or reduce automation that might coerce and manipulate learners’ thinking, impeding rather than supporting their motivation and identity development.

The focus of the previous dimension may be defined as the autonomy of will (Caughey et al., 2009) or positive freedom, referring to the capacity to develop one’s independent wishes and intrinsic motivations. Nevertheless, autonomy also underpins the autonomy of action (Möller, 2009), which refers to the ability to act on preferences without external restrictions. The AIED system relies on vast amounts of data to make predictions, which, in many cases, results in undesirable deductions of options to prevent users from engaging in or performing actions that the system views as errors (Bryson & Theodorou, 2019). Facebook’s decision to change its algorithm to prevent fake news and use fake IDs is one example. These interventions from AI, regardless of best intentions, potentially limit individual freedom of expression (an identity) or perform certain actions. Therefore, Fagan & Levmore (2019) suggested that humans ought to remain in the center of AI design and implementation, to be the ones presumably deciding the goals of AI and have the power to overrule machine decisions.

Of all AIED, a tool for assessing and providing guidance for students, predominantly referred to as an “intelligent tutoring system”, is the longest researched and most common application (Miao et al., 2021). The system mapped out learning materials and activities based upon experts’ knowledge of the subject and cognitive sciences, as well as student misconceptions and success. With increasing automated decisions and shortcut suggestions made by machines, it is likely that AIED will reduce learners’ interaction with others and their ability to cultivate individual resourcefulness, metacognition, self-regulation, and independent thought. One of AIED’s main ethical concerns is the possibility of undermining learner agency and pertaining to breaching autonomy of action.

To ensure a human-centric AIED that emphasizes the learner agency, researchers, developers, and practitioners must adopt an interdisciplinary approach to developing negotiation-based adaptive learning systems that emphasize but are not limited to transversal competencies (European Parliament, 2021). AIED should allow learners the power to negotiate the type and frequency of received support, scaffolding of not only knowledge but also metacognition and self-regulation skills (Chou et al., 2018; Daradoumis & Arguedas, 2020). Governments and educators should be aware of the AI literacy skills crucial for effective human-machine collaboration to develop and integrate the appropriate curriculum into education practices. As a result, not only will students and teachers remain in control and at the center of AI implementation, but humans and machines will also collaborate for improved educational outcomes rather than using AI to usurp humans (Bryson & Theodorou, 2019).

## Final remarks and future directions

The education system faces a paradox of artificial intelligence. Though regarded as vital for AI generation of high-quality educational outcomes, AIED and related large-scale collection and analysis of personal data about learners are of considerable concern to human-rights advocates. This paper contributes to the discussions of the benefits of AI in education, and at the same time, raises concerns for the adverse impacts on fundamental issues surrounding human rights. The intricacy of AI necessitates a holistic and applicable set of ethical principles for AI in the educational context. By systematically analyzing well-documented general AI principles, we propose a set of ethical tenets for AIED as a starting point to engage and spark further debates on the robustness of these guidelines, followed by actionable and shared policies to ensure the AIED systems developed are essentially ethical by design. The proposed set of ethical principles should be considered when developing and implementing ethical and trustworthy AI systems for education.

Nevertheless, given the growing interest of AIED in a post-Covid era, it is foreseeable that this debate concerning will continually evolve and move forward in the long run. A natural progression to be witnessed within the literature, namely a precise mechanism of ethical principles in AIED, remains to be elucidated. Indeed, while educational practitioners and AI developers have the best intentions of developing and implementing AI to improve education, the guiding ethical principles for AIED are yet to be set in stone. Furthermore, the education system has been confronting a paradox of applications of artificial intelligence technology across teaching and learning contexts. Despite existing theoretical frameworks investigating ethics of AI in general, no universal consensus has been reached on the best ethical theory in general, with moderate attention given to a practical set of ethical standards in the field of education in particular. Additionally, though regarded as vital for AI generation of high-quality educational outcomes, AIED and related large-scale collection and analysis of personal data about learners are of considerable concern to human-rights advocates. Such challenges call for greater attention in effectively and appropriately addressing associated ethical dilemmas. Given the interdisciplinary nature of AI, this is anticipated to be an arduous task to achieve since ethical principles are basically derived from human being judgement which can be largely abstract-driven, frequently inextricably intertwined with subjective interpretations. The engagement of diverse stakeholders in any educational discourse further impedes ethical principles from being widely applied either in a formal or deductive manner. Hence, based on our findings as preliminary ground, future scholarship is encouraged to extend the focus of inquiry to the implementation stage, where the issues of accessibility assurance, bias and equity in adopting AIED, or developmental and neurological influences of AIED to vulnerable groups such as young children and handicapped would be another interesting sphere that deserves continued exploration. Considerably more work will need to be done to establish and validate a common understanding and standards on ethics in AIED. A natural progression of this work is to publish a website about this set of ethical principles for AIED in order to capitalize feedback and improvement suggestions about the use of this framework. Furthermore, an automatic method by text analysis could be conducted in further work to provide complementing findings. Last but not least, embedding the principles of AI ethics in education, and also ethics issues such as responsibility, inclusion, fairness, security and explainability in conducting educational research will not only mitigate emerging societal abuses rooted from algorithmic injustice, but also bear instrumental implications to the landscape of AI governance and policy making for the long-term development of significant industries. Among one of the first papers to spark on the applicability of a set of ethical guidelines and practice standards for artificial intelligence in education, it is also our expectation that this will be a fruitful step towards guiding future educators and learners to exercise, if not instill, stronger accountability and responsibility in adopting AI and the technology that they employ for their teaching and learning in the future.

Overall, this paper contributes to the ongoing discussions of the benefits of AI in education, and at the same time, raises concerns for the adverse impacts on fundamental issues surrounding human rights. The intricacy of AI necessitates a holistic and applicable set of ethical principles for AI in the educational context. By systematically analyzing well-documented general AI principles, we propose a set of ethical tenets for AIED as a starting point to engage and spark further debates on the robustness of these guidelines, followed by actionable and shared policies to ensure the AIED systems developed are essentially ethical by design.

## Data Availability

Data sharing not applicable to this article as no datasets were generated or analysed during the current study.
